# Initiative to Improve Evidence-Based Chronic Obstructive Pulmonary Disease Hospitalist Care Using a Novel On-Line Gamification Patient Simulation Tool: A Prospective Study

**DOI:** 10.3390/healthcare9101267

**Published:** 2021-09-26

**Authors:** Jodi Strong, Larry Weems, Trever Burgon, Jeremy Branch, Jenny Martin, David Paculdo, Diana Tamondong-Lachica, Jamielyn Cruz, John Peabody

**Affiliations:** 1 Novant Health, 2085 Frontis Plaza Blvd, Winston Salem, NC 27103, USA; jpstrong@novanthealth.org (J.S.); lweems@novanthealth.org (L.W.); jsbranch@novanthealth.org (J.B.); jfmartin@novanthealth.org (J.M.); 2 QURE Healthcare, 450 Pacific Ave, Suite 200, San Francisco, CA 94133, USA; tburgon@qurehealthcare.com (T.B.); dpaculdo@qurehealthcare.com (D.P.); dlachica@qurehealthcare.com (D.T.-L.); jcruz@qurehealthcare.com (J.C.); 3 College of Medicine, University of the Philippines, Manila, Metro Manila 1000, Philippines; 4 Department of Epidemiology and Biostatistics, University of California, San Francisco, 550 16th St, San Francisco, CA 94158, USA; 5 Fielding School of Public Health, University of California, Los Angeles, 650 Charles E. Young Dr. South, Los Angeles, CA 90095, USA

**Keywords:** COPD, hospital medicine, clinical vignettes, care variation, quality, quality improvement, quality measurement, gamification

## Abstract

Chronic obstructive pulmonary disease (COPD) remains a leading cause of morbidity and mortality. Much of the disease burden comes from exacerbations requiring hospitalization. Unwarranted care variation and divergence from evidence-based COPD management guidelines among hospitalists is a leading driver of the poor outcomes and excess costs associated with COPD-related hospitalizations. We engaged with Novant Health hospitalists to determine if measurement and feedback using fixed-choice simulated patients improves evidence-based care delivery and reduces costs. We created a series of gamified acute-care COPD case simulations with real-time feedback over 16 weeks then performed a year-over-year analytic comparison of the cost, length of stay (LOS), and revisits over the six months prior to the introduction of the simulated patients, the four months while caring for the simulated patients, and the six months after. In total, 245 hospitalists from 15 facilities at Novant Health participated. At baseline, the overall quality-of-care was measured as 58.4% + 12.3%, with providers correctly identifying COPD exacerbation in 92.4% of cases but only identifying the grade and group in 61.9% and 49.5% of cases, respectively. By the study end, the quality-of-care had improved 10.5% (*p* < 0.001), including improvements in identifying the grade (+9.7%, *p* = 0.044) and group (+8.4%, *p* = 0.098). These improvements correlated with changes in real-world performance data, including a 19% reduction in COPD-related pharmacy costs. Overall, the annualized impact of COPD improvements led to 233 fewer inpatient days, 371 fewer revisit days, and inpatient savings totaling nearly $1 million. Engaging practicing providers with patient simulation-based serial measurements and gamified evidence-based feedback potentially reduces inpatient costs while simultaneously reducing patient LOS and revisit rates.

## 1. Introduction

Chronic obstructive pulmonary disease (COPD) remains a leading cause of morbidity and mortality both globally and in the United States [1,2,3]. In the United States, around 6% of adults over the age of 40 are living with this diagnosis [4]. Much of the disease burden related to COPD is due to disease exacerbations requiring hospitalization, with an estimated nearly 700,000 hospital discharges for COPD occurring in the United States alone [5]. Additionally, COPD-related hospitalizations are four times costlier than non-COPD hospitalizations [6].

The clinical and economic burden of COPD exists in spite of the established guidelines for improving COPD management. The Global Initiative for Chronic Obstructive Lung Disease (GOLD) guidelines were developed in 1997 and have been updated annually to help provide healthcare providers with the best evidence-based recommendations for the appropriate diagnosis and treatment of COPD patients [7]. However, despite the presence of these guidelines, recent studies have shown wide variations in care [8]; misdiagnosis—both underdiagnosis and overdiagnosis; and the incorrect treatment of COPD [9,10,11]. In the United States, for example, underdiagnosis occurs in roughly 12% of COPD patients [12] and overdiagnosis occurs in 48% of COPD patients [13], both of which can lead to excess healthcare expenditure through suboptimal management. Another study found that over half of the cost for inhaled medications due to COPD was wasted due to overdiagnosis and overtreatment [10].

To further system-wide goals to raise the standard of evidence-based COPD care, improve quality outcomes, and reduce waste, the Novant Health Hospital-Based Medicine Institute partnered with QURE Healthcare to provide their hospitalists with a patient-simulation based quality improvement tool. The focus of this project was an extension of the original QURE-Novant Quality Project (QNQP), which was aimed at heart failure and sepsis/pneumonia. The first project decreased practice variation, increased the quality of care provided, and realized savings of over one million dollars for the Novant Health system [14].

In this follow-on study, we extended this work by introducing a novel version of the engagement tool called QualityIQ for COPD patients. While similar to the Clinical Performance and Value (CPV^®^) vignettes used in the original study, QualityIQ is a novel version of CPVs explicitly designed to be stand-alone, mobile-ready, and easier to scale, delivering immediate evidence-based feedback to providers. In addition, the QualityIQ tool gamifies the learning experience by offering all providers an evidence-based quality score and showing how their care decisions compare to those of their peers with case-specific and overall leaderboards. 

In the last decade, there has been an increasing integration of datafication and gamification into medical and post-graduate medical education. The datafication of care, which is the process of converting phenomena into a quantified format so that it can be tabulated and analyzed [15], has been shown to increase the use of performance indicators and quality metrics among provider and healthcare system systems. We added the gamification element based on emerging evidence that this might further encourage providers to improve their care. Gamification, we hypothesized, will take advantage of the fact that humans are competitive, inherently playful, and find satisfaction in play [16]. Our hope is that gamification will result in a quicker and more effective acquisition of new knowledge, skills, and consistent clinical practice [17]. The gamified approach has been shown in other settings to be at least as effective as conventional approaches to skill instruction in ultrasonography [18] and has been proven to be an acceptable platform in internal medicine residency training [19]. In our own nation-wide pilot study where we first used the QualityIQ tool and gamified comparisons, the online care decision feedback game improved the performance of self-reported MIPS-based interventions in diabetes, asthma, and osteoarthritis cases [20]. 

Herein, we describe the results of the QualityIQ patient-simulation based measurement and feedback tool among practicing hospitalists caring for COPD patients and document the overall changes in quality of clinical decision making as measured by our tool and how this translates into rapid clinical and economic improvements in the real world.

## 2. Materials and Methods

### 2.1. Setting and Participants

Under the direction of the Novant Health Hospital-Based Medicine Institute, the 15 hospitals in the Novant Health system in North Carolina and Virginia were invited to enroll their hospitalists in the project. All 15 hospitals participated, for a total of 187 physicians and 58 advance practice providers (APPs). The participants included both employed and contracted hospitalist groups. The participation of providers was voluntary, and the participants received Continuing Medical Education credits and, if applicable, Maintenance of Certification points through the American Board of Internal Medicine for completing their cases. The employed providers had a small emolument tied to participation via an established incentive program.

### 2.2. QualityIQ Patient Simulation Cases

**Figure 1:**
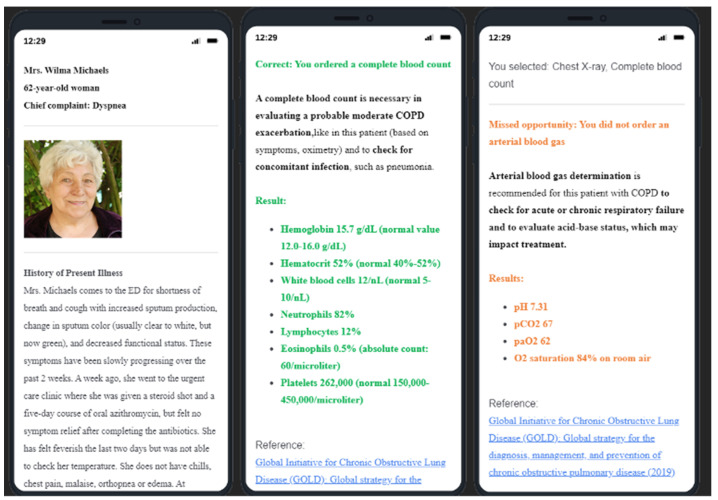
Screenshots of QualityIQ patient simulation case using mobile device. Note: No real-world patient data or images were used to generate the case simulation depicted here.

For this study, we created eight online QualityIQ case simulations, each of which presented typical work-up, diagnosis, and treatment decisions for a COPD patient in the acute care setting. As providers progressed through their online cases, they were given real-time evidence-based feedback. These eight cases and patient types are summarized in Table 1; screenshots of the mobile version are shown in Figure 1. Each case was a self-administered survey hosted on the Qualtrics Core XM platform, available from any internet-connected smartphone, tablet, or computer. The cases were designed to be completed in less than 10 min. Participants were given one new case every two weeks over the course of 16 weeks.

The QualityIQ simulated patients are online clinical cases. The cases recreate a typical clinical encounter by presenting initial history and physical exam finding and then asking providers to order their preferred diagnostic tests, identify the primary diagnosis and disease severity, and determine management and follow-up plans. Each domain is composed of 2–4 multiple-choice questions that provide options that a hospitalist would expect to consider in regular practice. After providers make their selections, correct and incorrect choices are revealed along with concise, evidence-based feedback providing rationale and supporting references for the recommendations. The feedback rationale is designed to be readily applied to similar scenarios in actual practice. The QualityIQ patient simulation cases are modified and mobile-ready versions of clinical performance and value (CPV) vignettes, which were designed and developed by QURE Healthcare and used successfully with the Novant Health group to support real-world practice change, are provided [14]. CPVs have been validated against actual clinical care and in numerous clinical settings [21,22,23]. CPV-driven initiatives have also been shown to reduce the cost of care (projected as $5 to $6.2M) and improve patient outcomes, such as length of stay and mortality [24,25].

While each case simulation was unique and required different care decisions to be made based on each patient’s presenting symptoms and risk factors, many of the care decisions were featured in multiple cases. The selection of the overlapping care decisions was identified by a Novant Health clinical leadership group, using the Novant Health patient-level data to specify the high-priority opportunities for quality improvement and care standardization, including, for example, the documentation of a COPD diagnosis with spirometry (to increase diagnostic accuracy) and proper indications for pulmonology referral and pulmonary rehabilitation referrals (to reduce readmissions).

### 2.3. QualityIQ Scoring

For provider care decisions and real-time feedback, the latest Global Initiative for Chronic Obstructive Lung Disease (GOLD) Guidelines was used as a reference. The overall and domain scores for each case were based on 11 or 12 multiple-choice questions covering the diagnostic domains consisting of work-up and diagnosis and treatment consisting of the initial and follow-up management decisions. Each question had explicit evidence-based scoring criteria identifying each option as necessary, unnecessary, or appropriate but not required. Participants ‘started’ with 100 points in each case. They earned 10 points for correctly selecting necessary items and lost 10 points for selecting unnecessary items. No points were added or subtracted for appropriate items. After each question, physicians received real-time feedback on their care decisions, including the appropriateness of their decision, recommended alternative decisions, and supporting evidence-based references for the preferred care path.

### 2.4. QualityIQ Gamification

Participants were asked to select a pseudonym for the project that was used to track their performance on an anonymous group leaderboard, which was updated after each case. At the end of each case every two weeks, the participants received a detailed score report that included a summary of key evidence-based recommendations for their case, their personal score in the case, and how their care compared to that of their peers. The top scores for each weekly case and the highest scorers across the entire season were awarded a $20 e-gift card for Amazon.

### 2.5. Post-QualityIQ Survey

We administered a post-participation survey after the 16-week QualityIQ rollout. The survey asked participants to provide feedback on the quality and value of the educational material, its relevance to their practice, its ease of access, the length of the case, specific action items they intend to revise in their practice, and the points that they liked most and least about the project. During this post-activity survey, participants are given the opportunity to express their desire to receive CME credit and ABIM MOC points.

### 2.6. Patient Level Impact Comparisons

We compared the profiles of the COPD patients seen at the Novant Health acute care hospitals over the time period of the study. To do so, we compared the patient characteristics for COPD MS-DRG 190 and 191 patients. These two MS-DRGs combined accounted for 91.0% of all COPD admissions at Novant over the relevant time periods and were aligned with the patient types in the QualityIQ cases. To avoid making unwarranted seasonal comparisons, we compared the year-by-year rates between 2018 and 2019 in the winter, spring, summer, and fall months that could potentially influence COPD presentation and/or admission. This was achieved by calculating the observed/expected (O/E) rates for the 2018 baseline year and for the (a) pre-season (January through June 2019); (b) in-season (July through October 2019); and post-season (November 2019 through April 2020). After determining these O/E rates for three measures—risk-adjusted cost, length of stay, and revisit rate (post-admission ED visits/re-admissions)—we then calculated the year-by-year percent difference for each measure in each time period, which we refer to as pre-season, in-season, and post-season.

### 2.7. Analysis 

We analyzed three primary outcomes changes: (1) overall and domain-specific performance in evidence-based care decisions made after each round of the QualityIQ cases; (2) specific high-value COPD action items such as PFT documentation, pulmonary rehabilitation, and unnecessary testing (cultures, procalcitonin); and (3) patient-level outcomes and economic savings using anonymized, real-world Novant Health COPD patients data collected before, during, and after the administration of the QuailtyIQ cases and feedback. Chi-squared tests and logistic regression modeling were used for analyses involving binary outcome variables, and we used Student’s T test for continuous outcome variables. All analyses were conducted in Stata 14.2.

## 3. Results

### 3.1. Baseline Provider Characteristics

Over the course of the project, 245 Novant Health Inpatient Care Specialist (NHICS)-affiliated hospitalists (both physician and APP) from all 15 facilities voluntarily agreed to participate. Over half of the participants were male (56.7%) and aged 40–55 (53.9%) and over three fourths were physicians (76.3%) (Table 2). On average, these providers had 11.6 ± 8.2 years of clinical practice experience, seeing 73 ± 41 in-hospital patients per week. When questioned about the variation in care across the Novant Health system, 80.6% of providers rated it as “None”, “Low”, or “Moderate”, while 63.4% perceived NHICS’s focus on improving quality and lowering unnecessary costs as either “Good” or “Excellent”. 

### 3.2. Baseline Scores

In the first, or baseline, round, we found a tremendous amount of practice variation as measured against the evidence-based scoring criteria. The overall quality of care performance scores for all providers was 58.4% and the standard deviation was ±12.3%. Providers scored better in the diagnostic domain (64.0% ± 19.8%), although there was even greater variation here as compared to the treatment domain (56.0% ± 13.7%). Providers at baseline ordered 0.83 unnecessary diagnostic tests per case, for an average cost of $181 (Table 3).

We drilled down into specific performance items at the baseline. We first looked at the work-up domain, wherein, as indicated by the guidelines and Novant Health order sets, a chest x-ray was needed and ordered in 92.4% of cases, a complete blood count was needed in 75.1% of cases, arterial blood gas was needed in 60.9% of cases, and bedside spirometry was needed in 43.4% of cases (Table 4). In cases where a particular test was considered low-value because it was unlikely to change the diagnosis or course of treatment, 33.7% unnecessarily ordered a respiratory infection panel (Biofire), 25.4% ordered a rapid influenza test, and 21.3% ordered procalcitonin.

In the diagnosis domain, providers correctly identified a COPD exacerbation in 92.4% of cases. However, using the GOLD criteria, they only identified the correct grade in 61.9% of cases and the correct group in 49.5% of cases.

In terms of post-diagnostic treatment, there were several noteworthy findings at the baseline. Providers admitted their patients to the correct level of care (outpatient management, medical floor, or intermediate care depending on the case) in only 60.4% of the cases. In cases where providers should have admitted patients to the floor, for example, they ended up doing so less than half of the time (45.7%), with the rest admitting the patients either to intermediate care (27.6% of cases) or outpatient or observation (26.7% of cases). Where indicated by the guidelines, providers appropriately prescribed steroids in 94.2% of cases and referred patients for inpatient pulmonary rehabilitation in 73.8% of cases. In five of the eight cases, where antibiotics were indicated, providers ordered the correct antibiotics in only 45.1% of cases. 

Low-value treatment items were common at baseline. For example, providers unnecessarily ordered an antitussive in 52.0% of cases and ordered a pulmonology referral/consult in 41.3% of cases when the patient’s condition likely did not warrant such care. When discharging their patients, disposition to the correct level of care was low at 40.9%, with a sharp difference according to type of discharge. In cases where discharge to home with self-care was ideal, providers correctly discharged patients to home 73.9% of the time. In contrast, when discharging to home where home health nursing care was preferred, that recommendation was made only 50.7% of the time. Moreover, when the patient was an appropriate candidate for receiving hospice care upon discharge, that recommendation was only made 1.8% of the time compared to 78.6% of the time these same patients were discharged to home (with or without home health care) and 19.6% were discharged to an SNF.

### 3.3. Changes in QualityIQ Scores across 8 Rounds

Compared to baseline, the overall QualityIQ scores improved 10.5% (*p* < 0.001) by the eighth round or by the fourth and final month of the study. This improvement was roughly the same for the diagnostic domain (+13.7%, *p* < 0.001) and the treatment domain (+10.1%, *p* < 0.001). Unnecessary workup also improved, decreasing to 0.30 instances per case or a reduction of 63.9%, with an associated cost decrease of $34 and an expense reduction of 81.5%. Both reductions were significant (*p* < 0.001).

The use of necessary diagnostic workup tests increased significantly as well, with appropriate chest x-ray orders increasing by +4.7% (*p* = 0.028), CBC orders increasing by +19.7% (*p* < 0.001), arterial blood gas orders increasing by +24.9% (*p* < 0.001), and bedside spirometry orders increasing by +22.0% (*p* = 0.006). Perhaps equally as important, the use of low-value diagnostic tests decreased for unnecessary respiratory panel (−28.4%, *p* < 0.001), procalcitonin (−16.6%, *p* < 0.001), and rapid influenza tests (−11.1%, *p* = 0.012). 

When making the diagnoses, although only the increase in identifying the COPD grade was statistically significant, there was an overall trend of improvement in all three diagnostic categories, with +3.3% more identifying a COPD exacerbation (*p* = 0.147), 9.7% more correctly identifying the COPD grade (*p* = 0.044), and 8.4% more identifying the COPD group (*p* = 0.098).

After eight rounds, providers were also more likely to correctly admit patients to the floor +13.2% of the time (*p* = 0.045), with nearly all of this improvement coming from decreased unneeded admissions to intermediate care or higher. Neither the ordering of steroids (−1.3%, *p* = 0.571) nor correctly ordering antibiotics (+3.8%, *p* = 0.529) changed significantly, but referrals for pulmonary rehabilitation did (+11.1%, *p* = 0.005). The use of unneeded antitussives decreased significantly (−38.3%, *p* < 0.001), as did unneeded referrals to pulmonology (−33.3%, *p* < 0.001). We did not see a significant increase in correct discharge disposition, either overall (+6.5%, *p* = 0.172) or by correct discharge level, although discharges with home health increased by +8.3% (*p* = 0.165), with decreasing discharges to home with self-care making up most of the change. 

In the post participation survey, after they had finished all eight cases, the participants were asked if they had done something different in their practice based on their participation in the program. In total, 72% reported practice changes based on the feedback they received for the QualityIQ cases.

### 3.4. Patient Comparison

To determine whether the improvements measured by the QualityIQ tool translated into real-world practice, we compared the profiles of MS-DRG 190 and 191 COPD patients seen at Novant Health acute care hospitals over three time periods. We first determined that there were no major differences in patient age, admission rates, payer types, or co-morbidities were apparent over the three time periods (Table 5).

### 3.5. Impact in Patient-Level Practice before, during and after Introducing QualityIQ

To measure the impact of the improvements in QualityIQ scores and the self-reported practice changes, we looked at the year-by-year changes in risk-adjusted cost, length of stay (LOS), and revisit (post-admission ED visits/re-admissions) rates for the COPD MS-DRG 190 and 191 patients. To achieve this, we compared real-world clinical practice over three separate time periods: (1) a ‘pre-season’, which was the 6-month period before the introduction of QualityIQ to the Novant Health Hospitalist (January 2019 through June 2019); (2) a ‘QualityIQ season’, when cases and feedback were actively administered (July 2019 through October 2019); and (3) a ‘post-season’, which included the 6 months after the QualityIQ season (November 2019 through April 2020). The comparison seasons matched the calendar months of the three seasons but occurred a year before—e.g., January 2018 through to June 2018 for the pre-season.

### 3.6. Clinical and Economic Benefit

**Figure 2:**
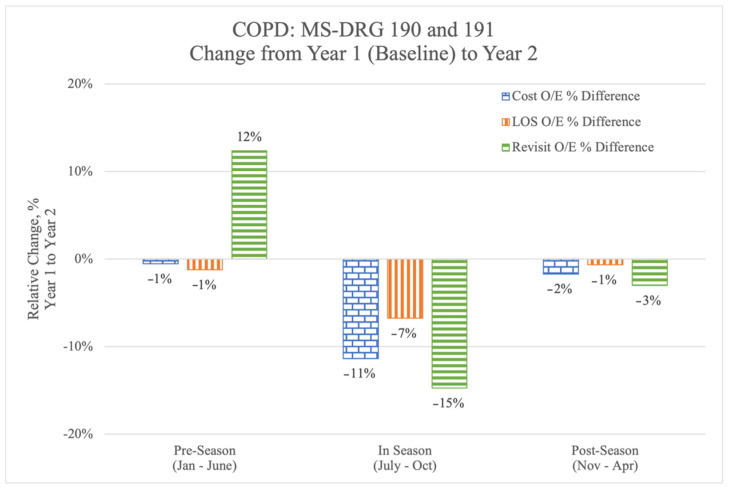
Year by year change in observed compared to expected cost, length of stay (LOS), and revisits by QualityIQ season.

In the pre-season, before the initiation of the QualityIQ program, the risk-adjusted year by year cost and LOS figures were relatively flat but revisit rates had increased by 12% (Figure 2) compared to 2018. During the 4 months of the QualityIQ program, risk-adjusted year-over-year cost, LOS, and revisit rates dropped dramatically and consistently. After the program ended, the measures in questions were lower than in the pre-season, although there was some regression from the time when the cases and feedback were being actively administered.

We next calculated the per-patient differentials (Table 6) using the annualized total per-patient costs for MS-DRG 190 and 191 patient populations at the Novant Health hospitals in 2019 (*n* = 1635). System-wide performance in the six-month pre-season prior to initiating the QualityIQ program would have resulted in an annualized increased cost of $9300, with 65 additional hospital days and 171 additional re-visit days to the hospital versus the 2018 values. By comparison, the annualized impact of Novant Health hospitalist performance during the QualityIQ season resulted in $964,952 in savings, 233 fewer inpatient days, and 371 fewer re-visit days. The post-season numbers sustained many of these improvements, leading to savings of $134,554, 32 fewer LOS days, and 75 fewer re-visit days. 

**Figure 3:**
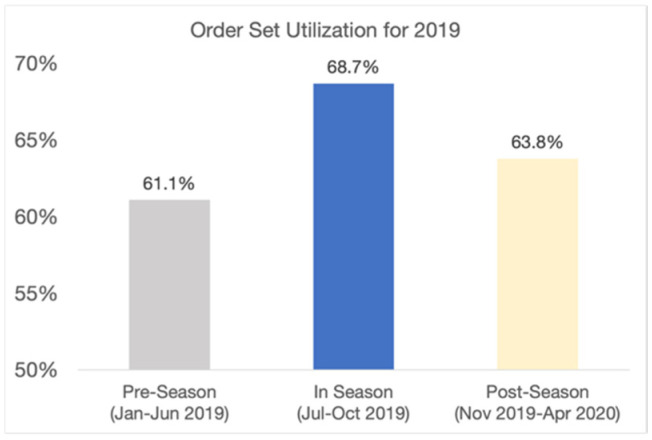
Order set utilization, 2019–2020.

**Figure 4:**
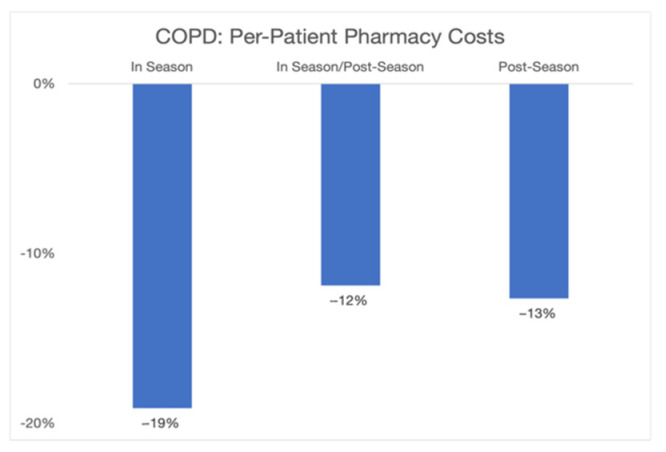
Year by year per-patient COPD pharmacy costs by season.

The overall economic savings of nearly $965 k came from reductions in LOS and re-visit rates and from better clinical care. To examine how the changes in clinical care contributed to lower costs and greater efficiency, we examined two specific areas: order set utilization and pharmacy resource utilization. The Novant Health COPD order set, available within the Novant Health electronic health record (EHR) and explicitly referenced in the QualityIQ provider feedback, is based on evidence-based guidelines and Novant Health best practices. The order set can be used to enhance the diagnostic work-up, treatment, and follow-up care for any COPD patient. We found that COPD order set utilization, as measured by Novant Health’s EHR system, increased dramatically during the QualityIQ season from 61.1% to 68.7% and then fell back to a mid-point in the post-season time period (63.8%) (see Figure 3). When we similarly examined changes in pharmacy costs for COPD patients, the year-by-year changes in pharmacy costs during the QualityIQ season decreased by 19% and those during the post-season period decreased by 13% (Figure 4).

## 4. Discussion

Improving the quality, efficiency, and value of COPD care is a primary focus for health systems, accountable care organizations, and payers across the country. COPD guidelines, for example, have been in place for years and are updated annually and used widely by practitioners. Notwithstanding, significant gaps in care remain. Even in a high-performing system such as Novant Health where the majority of providers rate care as good or excellent and less than 20% estimate care variability as high or very high, the gaps between guidelines and day-to-day practice commonly go unrecognized and new tools to close these gaps are urgently needed.

This study, which utilized the QualityIQ patient simulation-based engagement tool with real-time feedback on care decisions and gamified peer comparisons, is notable for several reasons. Over the course of eight serially administered QualityIQ patient simulations, practicing hospitalists were much more likely (18%, *p* < 0.001) to make correct, evidence-based care decisions for COPD patients. The hospitalist participants corroborated this finding, with 72% saying that they planned to make changes based on the personalized feedback they received in the QualityIQ cases. 

Impressively, these practice changes were manifest in real-world, patient-level data. Using longitudinal data independently generated by Novant Health, we observed shorter lengths of stay and lower pharmacy costs, saving nearly $1 million in risk-adjusted annualized costs. These quality and cost improvements were confirmed by other real-world data that showed the greater use of the COPD order sets and reduced COPD pharmacy costs. There are likely to be other significant savings made due to better care in other areas, such as fewer low-value tests and fewer complications, which we were unable to capture and quantify in the patient level data.

The clinical decisions measured in the QualityIQ cases provide clinically detailed insights into where the improvements occurred and where the remaining gaps in guideline-based COPD care persist. Providers, for example, frequently ordered low-value tests for COPD patients at baseline, including respiratory panel testing, procalcitonin, and influenza testing. Through serial measurement and feedback, the ordering of these low-value tests decreased by nearly two thirds (−63.9%). While cost reduction efforts typically focus on reducing unnecessary care, our results indicate that increasing evidence-based care items may involve providing more care that still drives efficiency and overall cost reductions. For example, the QualityIQ cases saw increases in the appropriate ordering of arterial blood gas, CBC, and bedside spirometry tests, supporting the argument that relatively modest increases in work-up costs can lead to large savings in the overall treatment through more accurate diagnoses being made and more appropriate treatment plans being drawn up. In addition to work up, we saw improvements in other areas, such as appropriate admission level and referrals to pulmonary rehab. Some gaps in care remained, however, and suggest productive areas for future quality improvement efforts. For example, while discharge disposition improved, particularly for home health referrals, appropriate referrals to hospice care for patients with advanced COPD with a desire to prioritize comfort over life-extending therapy were quite low at baseline. While these rates increased to 10% over the course of the study, additional improvement is needed and there is an opportunity to improve patients’ quality of life and reduce unnecessary spending further. 

One of the most interesting findings of this study is that the impact of the program was most pronounced while the providers were actively engaged with the QualityIQ cases and feedback. After the providers had completed their eight rounds, the O/E real-world improvements were moderated but did not fall all the way back to the baseline performance levels.

Other groups have found that quality efforts can also improve COPD care. For example, an integrated management program for COPD saved $11,263/patient annually and reduced readmission rates [22], while the separate education and training of medical staff in the guidelines improved compliance [23]. A Plan–Do–Study–Act framework demonstrated the increased use of treatments such as smoking cessation, pneumococcal vaccination, and diagnosis of COPD by spirometry [24]. Our work extends this current knowledge by demonstrating that QualityIQ case-based engagement with instant feedback and gamification can lead to significant real-world improvements in a way that is also massively scalable and easily distributable across various, geographically distributed sites.

While we have shown a strong connection between the QualityIQ engagement and real-world practice improvement, there are several limitations to our work that are worth noting. First, the most dramatic improvements were seen while providers were taking the cases, and there was some reversion once the cases ended. The longer-term impact of this 16-week effort has not been studied. Another unanswered question is the optimal cadence of cases. We believe that practice improvement is dynamic and requires ongoing engagement with the QualityIQ tool. Short 10 min cases can be easily scaled across large systems and may be appropriate for regular “drip feeding” to providers to help them stay on top of the latest guidelines and maintain their high performance. Future studies will be needed to explore sustainability. Secondly, although COPD is treatable, it is not curable, leaving us to wonder if patient outcomes improved in the long term. Although these results are indicative of better acute care, we do not know if this translates into better aggregate outcomes over time [25]. We believe that extending the benefits of QualityIQ engagement to PCPs would improve COPD care and extend the benefits beyond the acute care setting, building on the latest research showing that the QualityIQ tool is effective in the primary care setting [16]. Thirdly, since we did not have a contemporaneous control group to compare our results against, we cannot rule out a secular change in the care provided, such as structural changes in care transitions or group awareness of practice improvement opportunities. Notwithstanding this, we found that the greatest improvement was seen while the providers were taking the cases and that there was some regression afterwards. This indicates a direct effect from participation and feedback. Finally, we do not know if these results are generalizable to other healthcare systems, although past work suggests that this case-based approach works across a multitude of care settings and clinical areas [16,18,19,20,21]. The Novant Health hospitalists had previously worked with QURE to successfully improve patient care, so it is reasonable to assume that these findings, using a newer version of the simulation tool, are robust.

## 5. Conclusions

Unwarranted clinical variation remains a significant challenge. Even with the availability of high-quality guidelines, such as the GOLD criteria, the high rates of variation in diagnosis and treatment were notable at baseline. This study shows that variation can be reduced and care quality improved when practicing physicians and APPs care for simulated cases with timely feedback given for just 10 min on a bi-weekly basis. Providers engaged in this gamified educational approach are more likely to make evidence-based work-up, diagnostic, and treatment decisions. The reduced variation and higher provider quality lowered the cost of care for their real-world patients. This type of quality and cost improvement effort is a scalable and time-efficient approach that holds tremendous promise for health systems, practices, and payers looking to improve care quality and reduce costs in high-priority clinical areas such as COPD.

## Figures and Tables

**Table 1:** Summaries of simulated patient cases.

Case Types	Variant A	Variant B
COPD exacerbation needing antimicrobials	Severe COPD exacerbation in acute respiratory failure needing non-invasive positive pressure ventilation (NPPV)	Acute COPD exacerbation from influenza needing NPPV
2. COPD with non-respiratory complications	Acute COPD exacerbation and glucocorticoid-induced hyperglycemia in a patient with diabetes	Severe COPD in acute exacerbation needing NPPV and paroxysmal atrial fibrillation
3. Symptoms suggesting COPD and other comorbidities	Obesity hypoventilation syndrome mimicking COPD	Dyspnea in a patient with known COPD and prior MI
4. COPD needing palliative care and advance care planning	Persistent symptoms in a patient with COPD GOLD Grade 4 Group D on maximal medical management and nightly BiPAP	COPD GOLD Grade 2, Group C with frequent exacerbations despite maximal therapy in a patient in a skilled nursing facility

**Table 2:** Baseline provider characteristics.

	Results
N	245
Gender	
Female	37.1%
Male	56.7%
No answer	6.1%
Age Group	
<40	34.6%
40–55	53.9%
>55	11.5%
Physician (MD or DO)	76.3%
Number of years in practice	11.6 ± 8.2
Number of patients per week	73 ± 41
Time spent teaching, %	9.5% ± 16.1%
Perceived Practice Variability	
None	4.9%
Low	28.0%
Moderate	47.7%
High	14.0%
Very High	5.4%
Perceived Focus on Quality Improvement and Cost	
Poor	3.7%
Fair	9.1%
Average	23.9%
Good	42.0%
Excellent	21.4%

**Table 3:** QualityIQ scores by round.

	1	2	3	4	5	6	7	8	*p*-Value
N	225	217	2l5	214	200	217	214	211	--
Overall, %	58.4 ± 12.3	63.4 ± 11.7	65.9 ± 12.9	68.0 ± 13.3	68.1 ± 12.4	66.8 ± 15.3	68.8 ± 14.7	68.9 ± 14.2	<0.001
Domains, %									
Diagnostic	64.0 ± 19.8	70.7 ± 17.3	71.4 ± 18.1	73.9 ± 18.0	74.6 ± 18.5	73.2 ± 18.6	75.7 ± 18.2	75.7 ± 18.7	<0.001
Treatment	56.0 ± 13.7	60.2 ± 13.5	63.6 ± 14.8	65.6 ± 15.7	65.4 ± 13.6	64.1 ± 16.7	65.9 ± 15.9	66.1 ± 15.8	<0.001
Unneeded Tests									
No. per case	0.83 ± 0.94	0.65 ± 0.87	0.55 ± 0.78	0.52 ± 0.83	0.43 ± 0.76	0.44 ± 0.71	0.42 ± 0.72	0.30 ± 0.60	<0.001
Cost per case	$181 ± $269	$140 ± $245	$95 ± $207	$97 ± $212	$79 ± $195	$51 ± $153	$57 ± $161	$34 ± $124	<0.001

**Table 4:** Changes in specific QualityIQ scoring items.

	Round 1	Round 8	*p*-Value
Workup			
CBC	75.1%	94.8%	<0.001
Arterial blood gas	60.9%	85.8%	<0.001
Chest x-ray	92.4%	97.2%	0.028
Diagnosis			
COPD exacerbation	92.4%	95.7%	0.147
COPD grade	61.8%	71.6%	0.044
COPD group	49.5%	57.9%	0.098
Other diagnoses (influenza or pneumonia)	59.2%	62.8%	0.646
Treatment			
Correct admission level	60.4%	66.4%	0.201
Admit to floor	45.7%	58.9%	0.045
Admit to intermediate care	76.2%	74.8%	0.815
Bedside spirometry	43.4%	65.4%	0.006
Referral for pulmonary rehab	73.8%	84.8%	0.005
Palliative care consult	83.9%	81.6%	0.755
Stop smoking	86.4%	75.3%	0.067
Follow-up visit with PCP	93.4%	94.2%	0.752
Correct discharge disposition	40.9%	47.4%	0.172
Discharge home	73.9%	60.7%	0.320
Discharge home w/home health	50.7%	59.0%	0.165
Discharge hospice	1.8%	8.2%	0.126

**Table 5:** Patient characteristics.

	1 August 2018–31 July 2019	1 August 2019–31 October 2019	1 November 2019–31 January 2020	1 February 2020–30 April 2020
Patient Profile				
Discharges	2043	418	596	486
Average age	70	68	70	69
Admitted as Inpatient	98%	96%	97%	99%
Payer Type				
Commercial	49%	52%	50%	54%
Medicare	44%	38%	41%	34%
Medicaid	6%	7%	7%	10%
Self-Pay	2%	3%	2%	2%
Co-morbidities				
Diabetes	33%	32%	33%	32%
Lung Cancer	6%	6%	4%	8%

**Table 6:** Per-patient and total year by year differential savings/added costs normalized to year 2 patient distributions.

	Per-Patient Differential			Differential if Applied across All 2019		
	Cost	LOS	Revisit	Cost	LOS	Revisit
Pre-Season (January–June)	$5.66	(0.04)	0.10	$9250.76	(64.66)	170.57
In Season (July–October)	$(590.18)	(0.14)	(0.23)	$(964,952.07)	(232.97)	(371.28)
Post-Season (November–April)	$(82.30)	(0.02)	(0.05)	$(134,554.29)	(32.29)	(74.86)

## Data Availability

No additional data are available for this study.
